# A review of antimicrobial stewardship training in medical education

**DOI:** 10.5116/ijme.59ba.2d47

**Published:** 2017-10-12

**Authors:** Sarah L. Silverberg, Vanessa E. Zannella, Drew Countryman, Ana Patricia Ayala, Erica Lenton, Farah Friesen, Marcus Law

**Affiliations:** 1 Faculty of Medicine, University of Toronto, Toronto, Ontario, Canada; 2 Department of Family and Community Medicine, Faculty of Medicine, University of Toronto, Toronto, Ontario, Canada; 3 Gerstein Science Information Centre, University of Toronto, Toronto, Ontario, Canada; 4 Centre for Faculty Development, Faculty of Medicine, University of Toronto at St. Michael's Hospital, Toronto, Ontario, Canada

**Keywords:** Antimicrobial stewardship, antimicrobial resistance, antibiotic prescribing, undergraduate medical education, postgraduate medical education

## Abstract

**Objectives:**

We reviewed the published literature on antimicrobial stewardship training in undergraduate and postgraduate medical education to determine which interventions have been implemented, the extent to which they have been evaluated, and to understand which are most effective.

**Methods:**

We searched Ovid MEDLINE and EMBASE from inception to December 2016. Four thousand three hundred eighty-five (4385) articles were identified and underwent title and abstract review. Only those articles that addressed antimicrobial stewardship interventions for medical trainees were included in the final review. We employed Kirkpatrick’s four levels of evaluation (reaction, learning, behaviour, results) to categorize intervention evaluations.

**Results:**

Our review included 48 articles. The types of intervention varied widely amongst studies worldwide. Didactic teaching was used heavily in all settings, while student-specific feedback was used primarily in the postgraduate setting. The high-level evaluation was sparse, with 22.9% reporting a Kirkpatrick Level 3 evaluation; seventeen reported no evaluation. All but one article reported positive results from the intervention. No articles evaluated the impact of an intervention on undergraduate trainees’ prescribing behaviour after graduation.

**Conclusions:**

This study enhances our understanding of the extent of antimicrobial stewardship in the context of medical education. While our study demonstrates that medical schools are implementing antimicrobial stewardship interventions, rigorous evaluation of programs to determine whether such efforts are effective is lacking. We encourage more robust evaluation to establish effective, evidence-based approaches to training prescribers in light of the global challenge of antimicrobial resistance.

## Introduction

Antimicrobial resistance results from a host of factors, including improper antimicrobial prescription, and is an emergent problem. The next generation of physicians must be prepared to face this dilemma as antimicrobial stewardship becomes increasingly vital in the treatment of patients in all fields. 

Traditionally, antimicrobial stewardship programs (ASP) have been initiated to address the issue of antimicrobial resistance. Education, as a mainstay feature of these programs, is considered essential to teaching the knowledge necessary for effective stewardship, and can influence physicians’ prescribing behaviour.^1^^-^^3^ Several educational interventions have been shown to improve antimicrobial prescribing practices and infection control.^4^^-^^7^ The majority of antimicrobial stewardship education has involved practicing physicians, specifically targeting prescribing habits with the goal of modifying their approach to antimicrobial prescription.^1^^,^^8^ Yet changing the behaviour of practicing physicians has proven difficult.^6^

Few studies have sought to understand the type or efficacy of teaching practices of stewardship principles at the undergraduate and postgraduate medical education levels, particularly at the undergraduate level.^9^ As a result, the literature surrounding how early antimicrobial stewardship training starts in a physician’s career, and whether teaching these principles from the outset is an effective strategy for combating antimicrobial resistance and lowering overall resistance rates remains unclear. To date, there have been few comprehensive evaluations of antimicrobial stewardship teaching programs and overall effectiveness of these training procedures in undergraduate medical institutions, and those that have been done are geographically restricted.^14^ Accordingly, the extent to which this teaching translates into clinical practice is unclear. While recent studies identify the presence of stewardship curricula, the effectiveness of these interventions remains sparsely evaluated.^14^^-^^15^ In light of clinical evidence, it is critical to understand the role that formal learning has to play in teaching prescription habits in order to change such behaviours. Furthermore, an understanding of the effectiveness of such teaching strategies is needed in order to understand its impact, if any, on clinical practice.

Despite the focus of ASP on physician education, antimicrobial prescribing is frequently designated to junior doctors and residents who may not have developed sufficient expertise on antimicrobials.^10^^,^^16^ Furthermore, medical students have expressed interest in learning more about antimicrobials and their appropriate usage.^11^^-^^14^^,^^17^^,^^18^ Many students recognize the growing problem of antibiotic overuse, and most would like more training in this area.^13^^-^^14^^,^^17^^-^^19^ Students with formal training on antimicrobial stewardship, for example through didactic lectures with infectious disease specialists, feel better prepared for the practice and more comfortable with their knowledge.^11^ However, studies suggest students predominantly have significant gaps in their knowledge regarding appropriate use of antimicrobials.^13^^,^^16^^-^^18^^,^^20^^-^^24^ A number of studies have documented efforts of ASP for both undergraduate and postgraduate medical trainees.^9^^,^^25^^-^^28^ Yet no consensus has been reached as to how to ensure students are invested in antimicrobial stewardship and how to ensure retention of this knowledge and clinical implementation. Teaching appropriate use of antimicrobials varies greatly between countries and programs.^9^^,^^14^^,^^29^ A greater understanding of how these needs for ASP education are being addressed is an important next step towards addressing rising global awareness of the need for such principles in practice.

Despite student demand for learning on the topic of antimicrobial stewardship, and a focus on antimicrobial stewardship teaching at the physician level, it remains largely unknown at to the level of education at which medical trainees are first exposed to these concepts, how such principles are taught, and to what extent such teaching translates into clinical practice. We examined the published literature from 1946-2016 on antimicrobial stewardship for undergraduate and postgraduate medical trainees, including interventional design, how interventions were evaluated, and the effectiveness of the teaching strategies.

We conducted a mapping review with the purpose of organizing what is known surrounding effective teaching on antimicrobial stewardship.^30^ The review focused on the degree to which such interventions were evaluated to be able to make suggestions for further intervention adoption in medical education and to identify future areas of research. Given the heterogeneity of existing ASP research with respect to intervention type and evaluation, such mapping was valuable for examining the flexible implementation of interventions at the curricular level.^30^ Our goal was to better understand the spectrum of teaching strategies employed in educating students on antimicrobial stewardship principles and practices, and to understand how such practices have been evaluated. By exploring this aim, stewardship programs and medical schools can use our findings to implement teaching methods that effectively educate medical students and result in lasting change in the clinical setting, thus helping inform future best practice and curriculum design. Furthermore, by exploring this aim, it is possible to discern trends in education practices, and identify gaps remaining in our understanding of how to educate trainees on antimicrobial stewardship in a way that results in lasting prescribing habits.

## Methods

We conducted a literature review to compile a summary of the current published research on antimicrobial stewardship training in medical education. Ethical approval for this study was not deemed necessary, as our study constitutes a mapping review and did not involve primary data such as patient data collection or analysis.^30^ A systemic map contextualizes a detailed systematic literature review and focuses on key characteristics of the literature, including study population and setting, and is therefore ideal for understanding the nature of ASP educational interventions in terms of trainee group and key intervention characteristics.^30^^,^^31^

### Search strategy

We searched Ovid MEDLINE (1946 to 2016) and Ovid EMBASE (1947 to 2016), to identify research, review, and opinion articles addressing antimicrobial stewardship education for undergraduate and postgraduate medical trainees. Search strategies were developed by two authors (APA, EL). The authors translated the search strategies using each database platform’s command language and the appropriate search fields. MeSH terms, EMTREE terms, and textwords were used for the search concepts of antibiotics, prescribing patterns, and medical education. The three concepts were combined with a Boolean “AND.” No search limits were applied. Articles in English captured in EMBASE and MEDLINE were included in the final searches, which were completed in December 2016. We endeavoured to identify potential studies that were not captured from the database searches, by combing the reference lists of relevant studies and reviews included in the full-text review. Grey literature was not included in the search.

### Article selection

**Figure 1:**
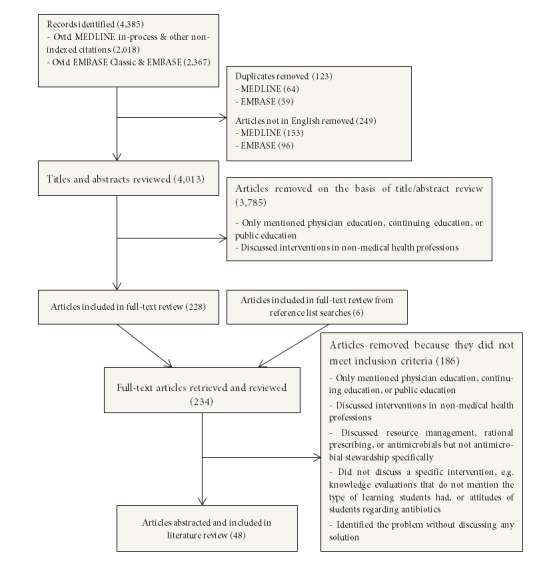
Search Strategy Flow Chart

Our database searches identified 4385 articles with potential relevance to our study. Three authors (SS, VZ, DC) identified the relevant articles for full-text review by examining the titles and abstracts of the articles identified in the database searches. We included articles written in English that discussed the use of antimicrobial stewardship in medical education. Articles that mentioned solely physician education, continuing education, or public education were excluded. Articles related to non-medical health professions education were also excluded as we focused on medical professionals in this review. Articles that discussed resource management, rational prescribing, or antimicrobials but did not discuss stewardship principles (for example, antimicrobial teaching focused on drug indications and dosing) were also excluded. Lastly, articles that identified only the problem (without discussion of any solution) or articles that did not discuss a specific intervention were excluded. Any disagreements between reviewers were brought to another author (FF), who resolved the dispute. After title and abstract screening, 227 articles were selected for full-text review. After the exclusion criteria were applied, 42 articles satisfied the inclusion criteria (see Figure 1). Searching the reference lists of the 42 included articles identified six additional studies for inclusion, for a total of 48 articles. For a complete list of all included articles, see Appendix A.

Each article included for full-text review was independently reviewed by two authors (two of SS, DC, VZ). The authors abstracted the following data fields from the included articles: authors, title, journal, year of publication, location, intervention, purpose of intervention, study design, participants, outcomes, key findings, and the level of evaluation from Kirkpatrick’s evaluation of education model:32 (1) reaction (satisfaction or happiness; what participants thought of the educational intervention), (2) learning (change in attitude and/or knowledge or skills gained assessed by test or demonstration), (3) behaviour (transfer of attitude, knowledge and/or skills to workplace or clinical setting, for example, determined through observation), and (4) results (patient care affected or societal impact due to participation in the educational intervention evaluated by looking at patient outcomes, for example).

## Results

Four types of articles were included in this mapping review: (1) commentaries/perspectives encompassing editorials and letters, some of which briefly discussed evaluations that had been done; (2) program descriptions without evaluation or outcomes measured; (3) review articles; and (4) research or evaluation articles with clear study design descriptions and outcomes. Across our dataset, there were nine perspective pieces,^25^^,^^33^^-^^34^^,^^39^^,^^41^^,^^44^^,^^47^^,^^60^^,^^68^ three program descriptions,^28^^,^^43^^,^^70^ five review articles,^42^^,^^45^^-^^46^^,^^52^^,^^71^ and 31 research articles.^9^^,^^15^^-^^18^^,^^24^^,^^29^^,^^35^^-^^38^^,^^40^^,^^48^^-^^51^^,^^53^^-^^59^^,^^61^^-^^67^^,^^69^

### Target learner group

Of the 48 articles included in this summary, 14 included only undergraduate medical trainees,^9^^,^^17^^,^^18^^,^^24^^,^^25^^,^^26^^,^^33^^-^^40^ 11 included both undergraduate and postgraduate trainees,^29^^,^^41^^-^^50^ 20 included only postgraduate trainees,^15^^,^^16^^,^^51^^-^^68^ and three did not specify the training level of the participants (see Table 1).^69^^-^^71^ The location of the studies also varied; the majority of studies were situated in Europe (20 studies; 41.7%), and 16 (33.3%) studies were based in the United Kingdom. Further, eleven articles (22.9%) took place in North America, three (6.3%) in Australia, eight (16.7%) in different Asian countries and two individual studies were conducted in South America and South Africa respectively. Four review articles included articles with geographical diversity.

### Intervention design

The types of interventions varied widely amongst studies and schools. Approaches at the undergraduate level (as demonstrated in detail in Appendix A.) included didactic teaching,^9^^,^^25^^,^^29^^,^^36^^,^^40^^,^^44^^,^^50^ web-based teaching,^39^ clinical case discussions,^35^ workshops/seminars,^26^^,^^34^^,^^69^ board games,^37^^-^^38^ guideline promotion,^35^^,^^49^^-^^59^ audits on current stewardship integration in the medical curriculum,^17^^-^^18^^,^^24^ and intensive modules,^47^ often in combination. Didactic teaching was moderately emphasized on the whole. Studies that examined educational interventions comparatively between medical institutions noted a widespread array of teaching strategies employed. These studies noted an overall inconsistency of trainees’ exposure to stewardship topics and of the intervention’s emphasis.^17^^-^^18^

Interventions aimed at the postgraduate level were similarly varied, and can be explored in Appendix A. These studies focused on providing feedback to students in relation to current prescribing practices rather than teaching new skills. Many methods provided resources for residents to consult and use to make prescribing choices and influence behaviours, or focused on identifying gaps in trainees’ knowledge and practice with the goal of rectifying their practice in the future. These approaches included: web-based tutorials,^47^^,^^67^ workshops,^57^^,^^62^^,^^65^^-^^66^ lectures and information sessions,^49^^,^^52^^,^^58^^,^^61^^-^^62^ using a restricted list of antibiotics to promote compliance with guidelines,55,58 audits followed by feedback,^15^^-^^16^^,^^52^^,^^56^^,^^60^^,^^64^^,^^67^^-^^68^ chart reviews and feedback,^51^^,^^58^^-^^59^^,^^66^ augmented reality,^53^ viral surveillance program,^61^ using special prescribing pads,^54^ integration of social media platforms such as Twitter and Facebook,^67^ consulting specialists to improve compliance with guidelines,^48^^,^^52^^,^^57^^,^^63^^,^^66^^-^^67^ and consulting guidelines or written information.^44^^,^^50^^,^^52^^,^^56^^-^^57^^,^^62^^,^^71^ Many interventions included a combination of these tactics. Interestingly, we found that auditing techniques evaluating prescribing tendencies was implemented at the postgraduate level, but not in undergraduate medical education settings.

**Table 1:** Articles that reported evaluations from each level of Kirkpatrick’s evaluation of education model, by intervention target group

Level of trainees	No evaluation	Kirkpatrick Level 1 (Reaction)	Kirkpatrick Level 2 (Learning)	Kirkpatrick Level 3 (Behaviour)	Kirkpatrick Level 4 (Results)
Undergraduate medical education	Davenport et al. 2005,^35 ^Kerr et al. 2001,^33 ^Luther, Ohl and Hicks 2013,^25^ Pulcini et al. 2015,^9^ Shankar et al. 2011,^26^ Wright and Jain 2004^34^	Beylefeld and Struwig 2007,^38 ^Chuenchom, et al. 2016,^ 17^ Haque et al. 2016,^ 18^ Hoque, Mostafa & Haque, 2016,^ 24^ Marwick and Nathwani 2007,^39^ Minen et al. 2010,^36^ Valente et al. 2009^37^	Huang et al. 2013,^40 ^Marwick and Nathwani, 2007,^39^ Valente et al. 2009^37^		
Postgraduate medical education	Brennan and Mattick 2013,^52 ^Philp, Wilford and Low 1986^51^	Bannan et al. 2009,^55^ Gharbi et al. 2016,^ 16^ Nifakos, Tomson and Zary 2014,^53^ Nand et al. 2016,^ 68^ Temte et al. 1999,^61 ^Welch et al. 2000^54^	Faryna, Wegowske and Goldenberg 1987,^56^ Feucht et al. 2003,^57^ Ikai et al. 2012,^58^ Pisano et al. 2016,^ 67^ Rawson et al. 2016^15^	Légaré et al 2011,^59^ Irfan et al. 2015,^65^ Main and Koerner 2012,^60^ McLellan et al. 2016,^ 66^ Temte et al. 1999,^61^ Welch et al. 2000,^54^ Zwar, Gordon and Sanson-Fisher 1995,^62^ Zwar et al. 1999,^63^ Lee et al. 2014^64^	
Mixed undergraduate and postgraduate medical education	Davey et al 1993,^29 ^Ghafur 2013,^41^ Greenwood 1998,^44^ Lee et al. 2013,^45^ Lee et al. 2015,^46 ^McNulty, Cookson and Lewis 2012,^43 ^Pulcini and Gyssens 2013^42^		Dawson, Bennett and Ongley, 2010,^47^ Zamin, Pitre and Conly, 1997^48^	De Souza et al. 2006,^50 ^Thamlikitkul et al. 1998^49^	Thamlikitkul et al. 1998^49^
Unspecified	Davey et al. 2007,^71 ^Le Normand et al. 1994^69^		Bain 1984^70^		

**Table 2:** Types of Interventions and their Kirkpatrick Level of Evaluation*†

Level of trainees	No Evaluation	Kirkpatrick Level 1	Kirkpatrick Level 2	Kirkpatrick Level 3	Kirkpatrick Level 4
Undergraduate	Didactic teaching (4) Workshop/seminar (3) Clinical case discussion (1) Guideline promotion (1)	Didactic teaching (1) Board game (1) Audit (3)	Didactic teaching (1) Web-based teaching (1) Board game (1) Intensive module (1)	Guideline promotion (2) Didactic teaching (1)	
Postgraduate	Specialist consult (3) Guideline use (2) Didactic teaching (1) Audit (1) Chart review (1)	Restricted antibiotic list (1) Augmented reality (1) Audit (2)	Guideline use (2) Specialist consult (3) Web-based teaching (2) Workshops (1) Didactic teaching (1) Chart review (1) Restricted antibiotic list (1) Audit (3) Social media & online education (1)	Didactic teaching (2) Audit (2) Guideline use (3) Specialized prescribing pads (1) Viral surveillance program (1) Specialist consult (3) Chart Review (3) Workshops (3)	Didactic teaching (1)

*Numbers reflect the number of different studies employing each intervention type

†Some studies used more than one type of intervention, or the same intervention in both undergraduate and postgraduate students, but specific interventions are listed separately

### Outcomes (variables of interest)

Studies measured a wide variety of outcomes (see Appendix A for full list of variables of interest). Although it was clear from the studies included in this review that medical students required supplementary antimicrobial education and desired their curricula to be augmented, all but one of the articles that measured outcomes (Kirkpatrick’s Levels 1-4) reported that the intervention made a difference. Notably, one exception we identified was an article by De Souza et al., which evaluated the current medical curricula from an ASP perspective, but did not involve a more specifically active intervention.^50^ Additionally, all studies that evaluated outcomes on a Kirkpatrick Level 1 scale supported sentiments echoed in the literature that, despite resources available in hospital or knowledge gained through the intervention, trainees do not feel comfortable with their knowledge. In studies evaluating behavioural change (Kirkpatrick Level 3-4), most outcomes included rates of prescriptions (total and/or inappropriate prescriptions), and fewer directly evaluated patient safety or cost parameters. In studies that evaluated knowledge (Kirkpatrick Level 2), there was variability between those evaluating students’ knowledge of stewardship principles and those evaluating the application of such principles to sample cases in a controlled setting.

### Evaluations

Over half (n=31, 64.6%) of the 48 studies included in this review reported that interventions were evaluated in some capacity. Eleven (22.9%) articles reported on a Kirkpatrick Level 3 or 4 (behaviour) evaluation of the intervention,^49^^-^^50^^,^^54^^,^^59^^-^^66^ eleven (22.9%) reported on a knowledge evaluation (Kirkpatrick Level 2),^15^^,^^37^^,^^39^^-^^40^^,^^47^^-^^48^^,^^56^^-^^58^^,^^67^^,^^70^ nine (18.8%) reported only an evaluation of participants’ reactions (Kirkpatrick Level 1),^16^^-^^18^^,^^24^^,^^36^^,^^38^^,^^53^^,^^55^^,^^68^ and 17 (35.4%) reported no evaluation of the intervention at all.^9^^,^^25^^,^^26^^,^^29^^,^^33^^-^^35^^,^^41^^-^^46^^,^^51^^-^^52^^,^^69^^,^^71^ Of those evaluating behaviour, no articles reported long-term behaviour changes after the intervention was completed, as only pre- and post-intervention evaluations were conducted. Of interventions that were evaluated, a mix between summative and mixed summative/formative evaluations was conducted.  None of the articles that targeted undergraduate medical students evaluated whether the intervention influenced their prescribing behaviour either as a senior medical student or followed them beyond graduation (see Table 1). No undergraduate evaluations were followed up by further postgraduate evaluations, and no undergraduate articles examined the longitudinal effect of the measured intervention on future practice.

Additionally, more individualized teaching methods, such as audits, consults, and chart reviews were more frequently evaluated at a higher Kirkpatrick level, while workshops and didactic teaching, interventions that often occurred in larger groups rather than one-on-one, were generally not evaluated at a high level (see Table 2).

## Discussion

In this review, we found a myriad of training approaches to antimicrobial stewardship in undergraduate and postgraduate medical education. While our understanding of antimicrobial resistance has grown, our understanding of effective educational approaches on the topic has not.  Approximately one quarter of educational interventions evaluated behaviour change (Kirkpatrick Level 3), and of these, only two articles that included undergraduate education evaluated interventions at this level. Further, studies frequently did not proceed to evaluate interventions at all. There continues to be a dearth of information available as to whether the effects measured by the studies that did evaluate their outcomes last beyond the immediate intervention time frame. The long-term effects of these educational interventions are unknown as the studies we identified largely do not include follow-up. It remains unclear whether these identified interventions are effective, more broadly, at influencing physician behaviour. In order to change the prescribing habits of future physicians and to lower antimicrobial resistance rates by teaching trainees best practices, more robust evaluations of innovative teaching strategies are needed. Many studies included in this review (n=17), did not evaluate the teaching strategies employed by respective investigators; instead, they simply reported on implementation. Evidence-based curriculum development is integral in the facilitation of best clinical practices. Medical educators and researchers must rigorously evaluate the efficacy of new educational interventions.^72^ Particularly in the undergraduate medical setting, it is invaluable to understand whether teaching about appropriate use of antimicrobials is retained by students as they go out to practice. We must understand whether interventions can be effectively applied at this level to influence prescribing behaviour on a long-term basis (i.e., Kirkpatrick Level 3), which will be instrumental in facilitating better patient care and societal impacts collectively (i.e., Kirkpatrick Level 4).

With antimicrobial resistance a growing global concern, there have been calls for further integration of teaching on this topic at the undergraduate medical level.^42^ Pulcini et al. identified physicians’ lack of knowledge of infectious diseases and antibiotics as a cause of their inappropriate prescribing,^28^ and both Pulcini et al. and Davey et al. recommended the translation of prescribing principles into learning topics and competencies for undergraduate core curricula.^29^^,^^42^ Our results support the findings of the Infectious Diseases Society of America that education on appropriate antimicrobial prescribing is highly variable across training facilities in the United States.^73^ In addition, similar inconsistencies in training seen on a global scale. Several recent studies conducted in the United Kingdom show that educational standards concerning antimicrobial stewardship vary greatly between postgraduate curricula, and provide further evidence that such inconsistent standards are a problem globally.^73^ To identify which of these educational interventions is most effective in bolstering medical student’s antimicrobial knowledge, our study shows that further evaluations of current approaches are needed. Once effective training programs are identified, such programs should then be expanded across undergraduate medical curricula. Recent changes to the Leader role in the CanMEDS 2015 physician competency framework, emphasizing quality improvement and patient safety,^74^ align with the appraisal of antimicrobial stewardship required at the undergraduate level. CanMEDS is a framework that outlines requisite competencies physicians need to successfully enact in order to meet the health care needs of the individuals and groups they serve. One of the competencies of the Leader role is that a physician must “engage in the stewardship of health care resources”.^74^ With this objective in mind, developing antimicrobial resistance education at the undergraduate level and evaluating such strategies to better educate future physicians fits well with the CanMEDS framework.

The educational interventions included in this review were highly varied, and different interventions were often used in different combinations. This practice of trying various strategies reflects the directive, led by Julio Frenk and Lincoln Chen, necessitating transformative and creative teaching initiatives.^75^ However, the included articles also span many decades and demonstrate little forward momentum towards improving and implementing ideas that have been shown to be promising in the past. While there is a great need for innovation and for the development of new ideas, there is no need to re-invent the wheel. More research must be executed to build on existing interventions and design effective learning programs. Collaboration between medical schools and the breakdown of institutional barriers (i.e. sharing best educational practices) can help accelerate this development process. Sharing resources and learning across institutions, as described by Frenk et al.,^75^ is integral to moving forward, but it also requires stringent evaluation metrics to assess collaborations and new educational interventions.

Finally, it remains unclear as to the extent that antimicrobial stewardship is actually incorporated into undergraduate medical curricula, particularly in different settings globally. Future research should target the extent and nature of these stewardship programs and must evaluate their overall effectiveness.

Due to the nature of our study, there are certain limitations to the interpretation of our results. Our review discusses the available English-language published literature, and does not capture studies that have been exclusively published in other languages. Thus, our findings might reflect the stewardship practices of the English-speaking world more heavily. However, considering the geographical diversity in the included studies, our findings are likely generalizable (on an international scale). In addition, we conducted our literature search through peer-reviewed journal articles indexed in two databases, excluding other databases. Other program descriptions and practices existing outside of peer-reviewed journal articles (e.g., grey literature or book chapters) were not included in this review; these bodies of literature are unlikely to include evaluations of antimicrobial stewardship interventions and were deemed outside of this study’s scope. However, the exclusion of grey literature from this review may limit our understanding of global trends due to the lack of published evaluation of such medical curricula. Finally, our review focused entirely on medical prescribers at the trainee level, and therefore the role of other health care professionals in antimicrobial stewardship practices was not included. These allied health professionals play a significant role in antimicrobial stewardship, particularly in jurisdictions where autonomous prescribing capabilities are authorized. However, comparing medical training with other health professional fields, and evaluating stewardship training in each profession’s curricula, are difficult to compare due to their vastly different structure and outside this study’s scope.

## Conclusions

Although our understanding of the scope of antimicrobial resistance has progressed, our understanding of which educational approaches to antimicrobial stewardship are effective has not. As physicians strive towards evidence-informed education, we need an evidence base that captures the impact of such programs on trainees’ prescribing behaviour after graduation. Currently, there are no best practice or national guidelines for teaching antimicrobial stewardship in undergraduate and postgraduate medical education. However, our study demonstrates that medical schools worldwide are supporting multitudinous antimicrobial stewardship interventions. It is possible that many intervention designs result in the desired outcome, simply by trainees’ attention to this area of interest. If so, there is an opportunity to optimize learning outcomes and intervention efficiency. Yet if such gains are short-lived and not habit forming, we must return to the drawing board to once again target prescribing habits in a new manner. Are we wasting resources and supporting ineffective interventions that are not cost-effective? Are we wasting time and trusting that these interventions will lead to a future culture of stewardship? Further rigorous evaluation of existing programs that capture Kirkpatrick Levels 3 and 4 are necessary to answer these questions, and thereby encourage additional medical education programs to incorporate such practices into their curricula. In order to foster a culture of stewardship amongst our trainees, we must develop and assess proficient educational interventions.

### Conflict of Interest

The authors declare that they have no conflict of interest.

## Supplementary material

Supplementary file 1 Appendix A. Studies with antimicrobial stewardship medical education interventions
