# Supplementary material for: A review of antimicrobial stewardship training in medical education
Source: Int J Med Educ. 2017 Oct 12;8:353–74. doi: 10.5116/ijme.59ba.2d47 (PMC5694692; doi:10.5116/ijme.59ba.2d47)
Supplement: Supplementary file 1 — Appendix A. Studies with antimicrobial stewardship medical education interventions [file ijme-8-353-S1.pdf]

Appendix A

Studies with antimicrobial stewardship medical education interventions

Author, year	Article type and location	Purpose of intervention	Intervention design	Target learner group	Variables of interest	Results and conclusions	Kirkpatrick evaluation level*
Brennan and Mattock, 2013	Review N/A	N/A	Intervention types include: educational materials, conferences and training, audit and feedback, outreach visits, reminders, marketing, patient-mediated interventions, and use of a local opinion leader. Most interventions used more than one strategy.	Residents and young doctors. Young prescribers in other health professions	A variety of outcome measures have been used, most commonly rates of appropriate/inappropriate prescribing	Most interventions were effective. No firm conclusions can be drawn about the most effective type of intervention, as some studies found one strategy effective while others found the same one ineffective.	No evaluation
Davenport et al, 2005	Research United Kingdom	To understand outcome-based education and how it has been applied and then been implemented in regards to antimicrobial prescribing	A resource template for teaching prudent prescribing included: (i) standardized vignettes covering the 12 learning outcome domains; (ii) a reflective patient record book, including a personal antibiotic formulary; (iii) discussion session guidelines; (iv) prescribing exercises; and (v) a support resource pack.	Medical students	Prescribing patterns	Educational approaches should strive to achieve predetermined outcomes and the intervention should be used in curricula	No evaluation
Davey et al, 2007	Review United Kingdom	To press for greater coverage of prudent antimicrobial use in undergraduate and postgraduate curricula; to lead in better integration of antimicrobial teaching in a variety of settings	Specialist Advisory Committee on Antimicrobial Resistance evaluating educational approaches	N/A	Attitudes and belief post ASP intervention through questionnaire, number of requests for restricted antibiotics	Hospitals continue to use different metrics in surveillance but are eager to collaborate. The SACAR Initiative has established a framework for UK collaboration on multi-disciplinary education that will define learning outcomes for all health professionals.	No evaluation
Davey et al, 1993	Research United Kingdom	To examine methods of determining and influencing antimicrobial use in the UK - specifically, evaluating undergraduate and postgraduate education on antimicrobial chemotherapy to establish what info is available and amount and content of the education	Questionnaires evaluating undergraduate and postgraduate curricula; undergraduate teaching mostly lecture based, postgraduate included consulting specialists and using material from drug information centres	Medical students and residents	What are the main factors that influence prescribing behaviour	The median amount of core undergraduate teaching was 13.5h but with a wide range. Content was oriented towards drugs rather than diseases. Most teaching was by formal lecture as part of a core programme. Postgraduate: advice from specialists and requests from practitioners most important determinants of content. Information from drug info centres discussed drugs not diseases. Knowledge	No evaluation

Author, year	Article type and location	Purpose of intervention	Intervention design	Target learner group	Variables of interest	Results and conclusions	Kirkpatrick evaluation level*
						should be disseminated through local networks of practitioners; all doctors require education; need national coordination of information dissemination	
Ghafur 2013	Opinion India	To reduce resistance rates and treat bacterial infections; to develop a functioning policy for antibiotics use and infrastructure for infection control	Curriculum must be revised to be less didactic, focus more on diseases of the tropics, rational antibiotics usage, and infection control.	Medical students and postgraduate trainees	N/A	Incorporate more infectious disease training in all major teaching hospitals; more clinically oriented basic science training	No evaluation
Greenwood, 1998	Opinion United Kingdom	Antimicrobial resistance is a growing problem that must be curtailed; in order for antimicrobial chemotherapy to have a secure future, prescribers must learn to use these tools with greater discretion	Teaching in the UK is spasmodic. Nottingham offers a 2-week module on antimicrobial therapy for third year students. Most schools: a few lectures on principles, and practical prescribing should be learnt at the bedside. Postgraduate: little is done.	Medical students and residents	Hours and types of hours spent in undergraduate and postgraduate settings	Students are still unprepared and are not knowledgeable in stewardship	No evaluation
Kerr et al, 2001	Letter to the editor United Kingdom	To understand the amount of contact time allocated to teaching about the rational use of antimicrobials at UK medical schools and the University of St Andrews	Research study described: postal survey	Medical students	Number of contact hours; methods of teaching	Number of hours dedicated varies greatly (0.5-22h); difficult to quantify exposure during teaching of other topics and in clinical setting; main methods of teaching was generally lectures, but also supplemented with tutorials and learning exercises, as well as self-directed learning	No evaluation
Le Normand et al, 1994	Research France	To develop a program to help teach the basic principles of antibiotic monitoring for time-dependent and concentration-dependent bactericidal	Multidisciplinary tutorial programs. Students given clinical case information, and plan treatment. System evaluates students' choice and provides commentary to evaluate the efficacy of treatment	Does not state	Not stated	Programs are complimentary to conventional teaching	No evaluation

Author, year	Article type and location	Purpose of intervention	Intervention design	Target learner group	Variables of interest	Results and conclusions	Kirkpatrick evaluation level*
		molecules					
Lee et al, 2013	Review N/A	To summarize and discuss various strategies to minimize antibiotic resistance	N/A	Medical students post-graduate trainees, staff	Courses in numbers and days, number of inappropriate prescriptions, number of duplicative gram-negative coverage with iv fluoroquinolones, duration of inappropriate use	Decreasing resistance requires involvement of students, staff, and the public. While much education has been targeted at staff and some at children, undergraduate training courses would be successful if the students are imparted with adequate knowledge, and trained in developing the right attitude and behavior. All health professions require teaching	No evaluation
Lee et al, 2015	Review N/A	To investigate recent studies on the effect of interventions for promoting prudent antibiotics prescribing,	N/A	Medical students and staff	Usage of diagnostic tests, choice of antibiotics, clinical outcomes (length of antibiotic treatment, length of stay, mortality)	There is no randomized control trial study assessing the effectiveness of educational programs for medical students. There is no study measuring the effectiveness of an educational program on prudent antibiotic prescribing for medical students	No evaluation
Luther et al, 2013	Letter to the editor United States	To increase appropriate antibiotic use	Antimicrobial stewardship pilot curriculum: didactic presentations and exam questions in USMLE format; clinical years have small-group activities	Medical students	N/A	N/A	No evaluation
McNulty, Cookson and Lewis, 2012	Program description United Kingdom	Examines what is currently being done in terms of antimicrobial stewardship in the UK / in Europe	Inclusion of hygiene and aseptic practice in the induction for the foundation years; infection control procedures are key competencies in second year; inclusion of infection control in appraisal for foundation year and specialist registrar trainees; infection control elements within the exams of Royal Colleges	Undergraduate, postgraduate, public, staff	N/A	The CMO is at the point where they are asking if it is indeed being taught / included at an undergrad and postgrad level	No evaluation
Philp, Wilford and Low, 1986	Research United Kingdom	To better define the scope of the failure for residents to prescribe rationally despite their lecture-based training	Research pharmacist examined ward prescription sheets and questioned residents about antimicrobial orders compared to labs if labs ordered	Residents	Number of antimicrobial prescriptions written; before or after labs; rational and appropriate prescription of antibiotics	Residents prescribed rationally when they had seen the labs but prescribed inappropriately if they did so before labs came back. Residents do not follow the methodology they were taught in class	No evaluation

Author, year	Article type and location	Purpose of intervention	Intervention design	Target learner group	Variables of interest	Results and conclusions	Kirkpatrick evaluation level*
Pulcini and Gyssens, 2013	Review N/A	To educate medical students on principles of microbiology, infectious diseases and clinical pharmacology, and on prudent prescribing of antibiotics	N/A	Medical students, children, junior doctors, staff	When should antimicrobial stewardship education start; who should educate; how should they be educated	Education should exist in medical school starting in year 3 of a 4 year program, not just in post-graduate setting	No evaluation
Pulcini et al, 2015	Research Europe	To educate European medical students; to determine antibiotic education in medical curricula	Questionnaire and interviews with lecturers to evaluate medical school curricula	Medical Students	Teaching principles for prudent antibiotic use in undergraduate curriculum; Association among curriculum, antibiotic use and/or rates of bacterial resistance at the country level	Prudent antibiotic use principles were taught in all but one school, but only 4/13 countries had a national programme. Interactive teaching formats were used less frequently than passive formats: clinical case discussions, active learning assignments, web-based server software learning platform, E-learning, role-play were used variably; lectures were used in all cases. Opportunities for implementation in curricula were variable. There was no significant association between the level of teaching of prudent antibiotic use and either prevalence of bacterial resistance, outpatient antibiotic use and total antibiotic use. Lack of time one of the main constraints preventing it from being included more heavily in curricula. There is wide variation even amongst students within the same country in terms of exposure to antibiotic prescribing principles.	No evaluation
Shankar et al 2011	Program description Nepal	To improve teaching of antibiotics to medical students	Problem-based learning used to teach pharmacology with 10 developed subject areas that students should learn in pharmacology	Medical students	N/A	Rational use of antibiotics has been embedded throughout a small group module. The module with modifications	No evaluation

Author, year	Article type and location	Purpose of intervention	Intervention design	Target learner group	Variables of interest	Results and conclusions	Kirkpatrick evaluation level*
						can be considered for inclusion in other medical schools	
Wright and Jain, 2004	Opinion United Kingdom	To review undergraduate medical education on AMR	Survey on knowledge of antibiotics and resistance were given to final year medical students before additional teaching. Spread teaching by medical microbiologists (tutorials)	Medical Students	Knowledge of antibiotics and of resistant organisms; no analysis given on interventions	Students do not always know the brand names of generic drugs; students had a good understanding of resistant organisms but overestimated antibiotics prescription rates in hospital. No analysis given on intervention	Level 2 of medical students, but no evaluation of the additional teaching
Bannan et al, 2009	Research Australia	To elicit attitudes of the ASP's aims, utility, educational value, effect on patient care and ease of use	Self-administered questionnaire, ASP program required receiving ID approval of restricted antibiotics, attitudinal and belief questions measured.	Interns, RMO and staff	Knowledge, attitudes and beliefs on the system; use of the system	98% of staff found ASP reasonable. Most believed it made teams think carefully about antibiotic choice. It was time-consuming and detracted from clinical duties. Intervention was believed by most to improve patient outcomes.	Level 1
Beylefeld and Struwig, 2007	Research South Africa	To improve medical microbiology teaching and students enthusiasm for it	Quiz-type board game	Medical students	Two questionnaire surveys, a focus group interview, direct observation and the nominal group technique	Game format effective in improving student engagement	Level 1
Cheunchom et al, 2016	Research Thailand	To identify knowledge, perception & attitudes concerning antimicrobial resistance & infection control among final year medical students	Questionnaire-based study among final year Thai medical students	Final year undergraduate medical students	Survey assessed student's perception and attitudes towards AMR, and knowledge of AMR/ infection control. Further educational activities and hospital structure were examined	Student's knowledge concerning AMR, appropriate usage and infection control was limited. Only half of students recognized the existence of an AMS program or infection control unit in their hospitals, highlighting poor access and gaps in current medical education at these institutions	Level 1
Gharbi et al. 2016	Research United Kingdom	To identify junior doctor's knowledge, attitude, behaviours concerning antimicrobial prescription so	Cross-sectional survey investigating junior doctor's antimicrobial prescribing practices & educational needs	140 Junior doctors, form 5 London hospitals	Survey assessed prescribing behaviour of junior doctors, prescribing education & support in the hospital	A significant proportion of junior doctors reported prescribing of antimicrobials in the context of low self-perceived confidence and knowledge and	Level 1

Author, year	Article type and location	Purpose of intervention	Intervention design	Target learner group	Variables of interest	Results and conclusions	Kirkpatrick evaluation level*
		as to identify key areas to address future educational programs			through a needs- based assessment. Multiple logistic regression examined factors associated with confidence prescribing Abx as a junior doctor	had difficulty in accessing aid. 5 specific areas needed to bolster junior doctors practicing in secondary care. Specific areas of educational need included: principles of antimicrobial prescribing, diagnosis of infections, clinical review of patients c infections, prescribing in context of AMR & laboratory testing/ test results	
Haque et al. 2016	Research University of Sultan Zainal Abidin, Malaysia	Assess medical students' knowledge regarding antimicrobial resistance and prescribing and to identify any gaps in education	Cross-sectional, questionnaire-based survey	Medical Students (year 3, 4 & 5)	Questionnaire consisted of 21 questions to assess: confidence in prescription, AMR knowledge, amount of training in undergrad studies and self-reported antibiotics usage	There is an educational gap between theoretical input and clinical practice in terms of AMR and antibiotics prescription. The majority of students (88%) stated desiring more training in their current curriculum concerning antibiotic selection and theory.	Level 1
Hoque, Mostafa, Haque et al. 2016	Research Chittagong, Bangladesh	To gain insight of medical students concerning antimicrobial prescribing and resistance, assessing readiness from education at private school in Bangladesh	Cross sectional, randomized, questionnaire based study	Medical students (years 3, 4 & 5 (clinical year in Bangladesh)	Survey assessed student's confidence in AMR & prescription practices, knowledge, amount of training received in curricula and self-reported practices of antimicrobial usage	Students had a lack of confidence in the context of AMR/prescription practices due to a knowledge gap in the proper selection of antimicrobials.	Level 1
Minen et al, 2010	Research United States	To understand medical students perceptions of their medical education; to identify gaps in medical education	Online survey on perceptions and attitudes of their training in antibiotics. Curriculum had pharmacology and an infectious disease courses that included antimicrobial treatment selection	Medical students	Student opinions and preferences on importance of antibiotics and resistance, feedback and teaching; where students access knowledge;	Student recognition of importance of topic and want more instruction. Medical school curricula should be expanded to provide additional training timed with their clinical rotations. Students have no clear	Level 1

Author, year	Article type and location	Purpose of intervention	Intervention design	Target learner group	Variables of interest	Results and conclusions	Kirkpatrick evaluation level*
					basic knowledge of organisms and resistance	preference for digital reference use for self-directed continuing education.	
Nifakos, Tomson and Zary, 2014	Research Sweden	To investigate educational approaches using the real physical context to enrich the educational on antibiotics prescribing	Augmented reality using a drug box	Residents	Design-Based Research Methodology: problem analysis, investigation of information that should be visualized for the training session, and finally the involvement of the end users the development and evaluation processes of the prototype.	Must further examine how augmented reality could contribute to the improvement of competencies among healthcare professionals and decrease of antibiotics resistance. The prototype needs further development; it had a high level of acceptance amongst students	Level 1
Priyasheelta et al. 2016	Research, letter to the editor United States	Evaluate medical trainees' knowledge & education regarding antibiograms	20-item survey	Postgraduate medical students (residents & interns)	Survey assessed resident/ intern's education surrounding antibiograms: comprised of knowledge-based questions (including antibiograms/ breaking point) & sources of education related to antibiograms	Study found that medical trainees receive limited education surrounding AMR/AM prescription – specifically, this study highlights that education- surrounding antibiograms is deficient & requires further coverage in curricula. Almost all residents/interns were familiar with the definition of an antibiogram, but only 62.2% correctly identified the definition of a 'breakpoint,' and 77.9% knew how to obtain their institution's antibiogram.	Level 1
Marwick and Nathwani, 2007	Letter to the editor United Kingdom	To improve appropriate antibiotic prescription	Promotion of local good practice guidelines, audits of knowledge and behaviour. An outcome based web-based program for teaching and reflecting on learning of antibiotics prescribing	Medical students	Student and staff feedback; student knowledge evaluated through exams	More clinically focused teaching through such web-based approaches can effectively improve students knowledge	Levels 1 and Level 2
Valente et al, 2009	Research Brazil	To integrate basic Bacteriology with mechanisms of action of antimicrobial	Board game to teach students about antibiotics and resistance	Medical students and pharmacy students (under-	Knowledge pre- and post-intervention; survey on whether students liked	There was an increase in the number of right answers and a decrease in the number of unknown	Level 1 and 2

Author, year	Article type and location	Purpose of intervention	Intervention design	Target learner group	Variables of interest	Results and conclusions	Kirkpatrick evaluation level*
		agents		graduate)	the game, and whether they found it an effective learning tool, etc.	answers. There were no significant differences between the courses.	
Welch et al, 2000	Research United Kingdom	To promote rational antibiotic and analgesic prescribing for non-inpatients attending an emergency department	Survey, intervention (prescription pad including a summary of clinical condition) reviewed by panel of experts and rated for appropriateness, followed by educational period, which included lectures, discussion groups, and guideline promotion. Survey and review were repeated	Junior medical officers	'Non-inpatient' prescriptions were collected before education and afterwards.	Not statistically significant improvement in antibiotic prescribing trends; use of guidelines did markedly increase. A method has been developed to educate JMOs on therapeutics in the ED that led to a trend in improvement in prescribing of antibiotics but little change in analgesic prescribing. The program was very well accepted by the JMOs	Levels 1 and 3
Temte et al, 1999	Research United States	To improve viral surveillance and improve family medicine residents prescribing of antibiotics	Viral surveillance program as well as detailed educational seminar on respiratory viruses. Chart review to evaluate antibiotics prescription rates and upper respiratory infections	Residents	Familiarity with system and evaluation of training in terms of knowledge provided and its usefulness; rates of antibiotic prescribing in senior residents	By PGY-3 year, 79% felt their education and training had provided adequate knowledge. Most knew about the surveillance program but only 42% pay attention to surveillance reports. 86% had obtained specimens for viral culture. Educational intervention, while did not set out to change physician behaviour, did have this effect.	Levels 1 and 3
Bain 1984	Program description United Kingdom	To assess the diagnosis/treatments for acute earache in children developed by a trainee before and after 6 months with a trainer	24 Trainers and their Trainees were asked to examine the case histories of 5 children with 24 hours of ear ache	Trainers and trainees (does not state level)	Prescription rates of different medications	Fall in trainees prescribing of antibiotics for otitis media (although not statistically significant), but this was correlated with an increase in prescribing of decongestant-antihistamines	Level 2
Dawson, Bennett and Ongley, 2010	Letter to the editor United Kingdom	To evaluate the use of e-learning modules on antibiotic prescribing	A mandatory induction module; a module on antibiotic prescribing	All staff, including residents and medical students	Use of e-learner package and effect on on-site training	The modules have enabled the junior doctors to become familiar with Trust procedures and IPC and antibiotic policies, and have reduced the time required for on-site	Level 2

Author, year	Article type and location	Purpose of intervention	Intervention design	Target learner group	Variables of interest	Results and conclusions	Kirkpatrick evaluation level*
						induction training	
Faryna, Wergowske and Goldenberg, 1987	Research United States	To understand the impact of therapeutic guidelines on outpatient prescribing patterns of internal medicine residents	A one-page set of treatment guidelines was designed by internal medicine staff and placed on the desks of their examining rooms. A pre and post intervention survey was collected	Residents	Antibiotic choice and appropriateness	Pre-intervention showed 50% of antibiotic choices were inappropriate, but there was no significant difference in pre- and post-intervention antibiotic choices. The more experienced a resident, the less appropriate the antibiotics prescription pre-intervention, but there was no difference after between experience levels. The guidelines did not result in a significant decrease in proportion of inappropriate antibiotic. prescriptions. More effective surveillance is needed in the area of outpatient antibiotic prescribing by residents; audits are a practical way to monitor prescribing behaviour	Level 2
Feucht et al, 2003	Research United States	To improve IV vancomycin and fluoroquinolone prescribing practices with guidelines. (ie: encourage early discontinuation of inappropriate use or duplicative gram negative coverage)	Monthly or semi-monthly conferences on antimicrobial therapy and resistance, as well as guidelines and current hospital practices. Antibiotic use monitoring by clinical pharmacist with consultation if use deemed inappropriate. Reprints of the guidelines were also placed in the medicine physician conference rooms.	Residents and staff	Number of metrics including number of inappropriate prescriptions, length of course, and number of antibiotic courses overall	The intervention decreases unnecessary duplicative regimens, number of courses overall of some antibiotics, courses over 5 days, and inappropriate prescribing. Appropriate educational intervention programs can alter prescribing patterns in residents, particularly in early discontinuation of inappropriate therapy and altering prescribing practices of fluoroquinolones.	Level 2
Huang et al, 2013	Research China	Purpose of survey: to get an overview of the students' understanding of antibiotics, and to compare the medical	Curriculum gives intro of infectious diseases. Antibiotic prescription is supervised by clinicians during clinical internship. Gave questionnaire on: knowledge of antibiotics, attitude towards antibiotic	Medical students	Knowledge of antibiotic use; attitude and public education on usage of antibiotics; behaviour of	Medical school did improve knowledge of antibiotic use; majority of students thought antimicrobial resistance was a problem in China and results from	Level 2

Author, year	Article type and location	Purpose of intervention	Intervention design	Target learner group	Variables of interest	Results and conclusions	Kirkpatrick evaluation level*
		students' and non-medical students' knowledge, attitude and behaviour towards antibiotic use	use, perception of public education and practice towards antibiotic use.		using antibiotics	antibiotic abuse and more public education is necessary. Medical students scored worse than non-medical students on behaviour towards use of antibiotics. Medical students got better as they progressed through their years indicating that the schooling improved their knowledge	
Ikai et al, 2012	Research Japan	To understand the effect of postgraduate education on physicians' adherence to the guidelines or on patient outcomes.	Systematic lectures and case-based discussions. A hospital-wide restricted formulary to decrease carbapenem. Analysed via chart review.	Residents	Appropriateness of antibiotics prescribed, reasons for inappropriate use	More frequent blood, sputum cultures and gram stains; less frequent use of broad spectrum antibiotics as initial empiric therapy; median length of stay was shorter after intervention; de-escalation from parental to oral was not significant; more cases were treated with oral abs.	Level 2
Pisano et al. 2016	Research United States	Study aims to augment reach & boost antimicrobial stewardship through utilization of social media	Pre- and post- intervention surveys – including 20 questions on antibiotics & infectious diseases and awareness of ASP initiatives, social media usage & attitudes surrounding antimicrobial resistance	55 internal medicine residents	Pre- and post-intervention surveys after the ASP social media intervention (which included trivia questions on antimicrobial resistance/ antibiotics prescription, links to teaching articles or internal content on ASP website – using Twitter & Facebook)	Study concluded that social media proved to be a valuable tool to reinforce ASP initiatives and education, while encouraging the use of ASP so as to promote Antimicrobial mindfulness in clinical practice. antibiotics knowledge scores increased following the social media education intervention, and access to ASP increased from 70% - 94%. Further, more IMRs indicated using the ASP clinical pathways more frequently post- intervention	Level 2
Rawson et al. 2016	Research United Kingdom	To investigate the coverage of AMS across UK clinical postgrad curricula & assess quality of education	Cross-sectional analysis & quality assessment	Postgraduate medical students (37 specialties; inter-specialty analysis performed)	37 Specialties assessed: topics & individual learning points relating to antimicrobial stewardship & resistance were	Overall coverage of antimicrobial stewardship & resistance is relatively poor across the majority of UK post-graduate clinical training	Level 2

Author, year	Article type and location	Purpose of intervention	Intervention design	Target learner group	Variables of interest	Results and conclusions	Kirkpatrick evaluation level*
					extracted for each specialty, and these were then quality assessed, followed by inter-specialty analysis	curricula, with little depth of learning enacted. Of the 37 specialties assessed, 2,318 topics & 42,527 learning points were identified – of these, 0.3% topics & 0.4% learning points were related to antimicrobial stewardship/resistance. In contrast, primary care, which is responsible for the highest proportion of antimicrobial usage, only had 0.15% antimicrobial stewardship/resistance learning points. On quality assessment, 60% of learning points required knowledge only, with no demonstration of behaviour in clinical practice.	
Zamin, Pitre and Conly, 1997	Research Canada	To assess the effect of introducing a route conversion program on the prescribing of antimicrobials for the treatment of respiratory tract infections and skin/soft tissue infections	General medicine wards at a tertiary care health centre had a route conversion program had the insight of infectious disease physicians and microbiologists, as well as pharmacists	Medical students post-graduate trainees, staff	Clinical and laboratory parameters related to the status of the infection compared with course of therapy; patient's ability to meet the criteria established in the guidelines; number of days of IV therapy	Even after program introduction, the number of days that IV therapy was continued, despite the appropriateness of oral therapy, was reduced. The program had a positive influence on antimicrobial prescribing behaviour in the population studied.	Level 2
De Souza V et al, 2006	Research Ireland	Medical school curriculum: to teach students proper antimicrobial prescribing. Overall: to evaluate factors that influence prescribing by non-consultant hospital doctors	Curriculum design / influences on young doctors that is discussed: the practices of senior colleagues on their teams; personal experience later in their career; undergraduate lectures; hospital guidelines	Medical students and postgraduate trainees	Processing and mastery of medical microbiology learning content, enjoyment derived from intervention, Qualitative evidence of positive affect generated by intervention	While undergraduate training in medical microbiology provided information on infections, it left interns insufficiently trained to make autonomous antimicrobial prescribing decisions. Formal undergraduate education on antimicrobial agents, rationale for antimicrobial use, existing hospital guidelines and concerns about	Level 3

Author, year	Article type and location	Purpose of intervention	Intervention design	Target learner group	Variables of interest	Results and conclusions	Kirkpatrick evaluation level*
						emerging resistance appear to be minor influences. Those trained in low income countries had greater knowledge and autonomy in their prescribing	
Irfan et al. 2015	Research Canada	Identify risk factors for unnecessary prescription and to assess impact of educational intervention focused on prescription of antibiotics	Quasi-experimental study, with a control group	Medical residents	Study compared baseline period of patients with positive urine cultures to educational intervention period to compare appropriate antibiotic prescriptions	Educational intervention was effective in the reduction of unnecessary antibiotic use – 52% of control interventions were inappropriately prescribed compared to 8% following intervention	Level 3
Lee et al, 2014	Research Canada	To optimize antibiotic use through trainee-led time-outs	While receiving monthly education on antimicrobial stewardship, residents adjusted patients' antibiotic therapy through twice-weekly time-out audits using a structured electronic checklist	Residents (medical students were on the team during the audit, but it is unclear whether they participated)	Cost savings, comfort with antibiotic prescribing, patient prescription rates	$70,000 savings associated with 1 hour of faculty time and 8 hours of resident time. 80% of residents adhered to the auditing program and believed it improved their comfort with antibiotics and provided clinical value. 1 in 7 patients had their antibiotics changed after the first audit. Rates of C diff decreased. No mention of medical students directly	Level 3
Légaré et al, 2011	Research Protocol Canada	To improve the use of antibiotics in family medicine treatment of acute respiratory infections	Web-based tutorial followed by interactive workshops; addressed clinical decision-making process regarding antibiotics treatment	Residents and staff	Proportion of patients reporting a decision to use antibiotics immediately	N/A	Level 3
Main and Koerner, 2012	Letter to the editor United Kingdom and Europe	To reduce levels of antimicrobial resistance	Audit; using a restricted group of antibiotics	Residents	Incidence of multi-drug-resistant microbes	Reduced incidence of multi-drug-resistant microbes; not enough evidence to expand	Level 3
McLellan et al. 2016	Research United Kingdom	Investigate whether & how structured feedback sessions can increase rates of appropriate	Participants randomized to intervention (pharmacist-led feedback sessions) and control (routine practice) 2 days/week for 6 months, mean normalized prescribing rates &	Junior doctors (post graduate residents) in their first year of	Researchers assessed normalized rates of prescription. Further, thematic	Study found that the Pharmacist-led feedback intervention augmented appropriate prescription patterns in junior doctors, by	Level 3

Author, year	Article type and location	Purpose of intervention	Intervention design	Target learner group	Variables of interest	Results and conclusions	Kirkpatrick evaluation level*
		antimicrobial prescribing by junior MDs	qualitative interviews	training	analysis of qualitative interviews was completed to gain insight into the feedback workshops	acting as a positive stimulus within a complex network of behavioural influences. The authors conclude that prescribing behaviour is adaptable and can influence best practice guidelines by reducing suboptimal antimicrobial prescribing	
Zwar, Gordon and Sanson-Fisher, 1995	Research Australia	To improve rational prescribing of antibiotics and benzodiazepines	Randomized control trial evaluating a seminar on rational prescribing focusing on skills and knowledge. Included: information including reference to guidelines, process skills and feedback on performance. Group discussion then held and feedback on performance given.	Residents	Rate of antibiotic prescription for all conditions; prescribing of antibiotics for URTI, choice of antibiotics for conditions including tonsillitis, sinusitis, acute otitis media, etc. Benzodiazepine prescription for all conditions	Group educational approaches to influence prescribing can be effective. Decrease in antibiotic prescribing, but often non-significant decreases in intervention group and increases in control. Further research is needed to assess whether prescribing behaviour is affected by feedback, guidelines, reminders, etc.	Level 3
Zwar et al, 1999	Research Australia	To examine effectiveness of an intervention to reduce antibiotic prescribing in GP trainees for undifferentiated upper RTI and improve antibiotic choice	Individual prescriber feedback, followed by further resources and further visits. Prescriber feedback and educational outreach also given.	Residents	Diagnostic behaviour	Antibiotic prescribing by the intervention group declined, while the control group increased. Prescribing in agreement with accepted guidelines increased in the intervention group, but decreased in the control group. Prescriber feedback and management guidelines influenced antibiotic prescribing and choice of antibiotic.	Level 3
Thamlikitkul et al, 1998	Research Thailand	To reduce inappropriate prescribing in a hospital setting	Information feedback and antibiotic guidelines through information sessions. Data from patients prescribed antibiotics was collected before and after educational intervention	Residents, staff, and final year medical students	Frequency of antibiotic use and their cost pre-and post-intervention	22% reduction in antibiotics use in in-patients; 23% in outpatients. Most antibiotics are prescribed by residents, GPs in outpatient clinics and final year medical students. The educational program was effective.	Level 3 and 4

*Kirkpatrick Levels of Evaluation: (1) reaction (satisfaction or happiness; what participants thought of the educational intervention), (2) learning (change in attitude and/or knowledge or skills gained assessed by test or demonstration), (3) behaviour (transfer of attitude, knowledge and/or skills to workplace or clinical setting), and (4) results (patient care affected or societal impact due to participation in the educational intervention evaluated).[32]
